# Nucleolar and coiled-body phosphoprotein 1 (NOLC1) regulates the nucleolar retention of TRF2

**DOI:** 10.1038/cddiscovery.2017.43

**Published:** 2017-09-04

**Authors:** Fuwen Yuan, Guodong Li, Tanjun Tong

**Affiliations:** 1 Research Center on Aging, Department of Biochemistry and Molecular Biology, School of Basic Medical Sciences, Peking University, Beijing, China

## Abstract

Telomeric repeat-binding factor 2 (TRF2) was reported to localize in the nucleolus of human cells in a cell cycle-dependent manner; however, the underlying mechanism remains unclear. Here, we found that nucleolar and coiled-body phosphoprotein 1 (NOLC1) interacted with TRF2 and mediated the shuttling of TRF2 between the nucleolus and nucleus in human 293T and HepG2 cells. Ablation of NOLC1 expression increased the number of nuclear TRF2 foci and decreased the nucleolar level of TRF2. Conversely, NOLC1 overexpression promoted the nucleolar accumulation of TRF2. NOLC1 overexpression also increased the number of 53BP1 foci and induced the DNA damage response. In addition, co-expression of TRF2 rescued NOLC1 overexpression-induced cell cycle arrest and apoptosis.

## Introduction

The regulation of gene function involves a series of processes including protein production, modification, and degradation,^1,2^ in which the regulation of protein distribution in different subcellular structures is one of an important way.^3–5^ The nucleolus is a eukaryotic subnuclear organelle that is responsible for ribosomal RNA (rRNA) transcription, processing, and modification and ribosome assembly.^6–8^ Accumulating evidence has linked the nucleolus with many other processes besides rRNA metabolism, leading to the concept that it is a plurifunctional organelle.^9–14^ Many studies show that the functions of some proteins are regulated via control of their nucleolar retention. For example, nucleolar proteins such as RPL11 can regulate the nucleolar retention of the E3 ubiquitin ligase mouse double minute 2 homolog (MDM2) and thus inhibit ubiquitination of p53.^15^ Other proteins such as RDM1, VHL, Hsp70, and PML also show a nucleolus/nucleus distribution regulation pattern.^16–18^ It was recently reported that telomeric components such as telomerase and TRF1 localize in the nucleoli of mammalian and yeast cells.^12,19,20^ In addition, the redistribution of silencing proteins from telomeres to the nucleolus is associated with lifespan extension in *Saccharomyces cerevisiae*.^21^ This reinforces the idea that the nucleolus participates in the regulation of telomeres.

Nucleolar and coiled-body phosphoprotein 1 (NOLC1) was first identified as a nuclear localization signal-binding protein and also functions as a chaperone for shuttling between the nucleolus and cytoplasm.^22,23^ The orthologues of NOLC1 in the human, Xenopus, Drosophila, and worm genomes ^24–27^ share a similar organization and contain conserved N-terminal and C-terminal domains and a central region consisting of several interspersed repeats of acidic and basic amino acid clusters. CUL3 monoubiquitylates NOLC1 together with TCOF1, thereby remodeling the translational program of differentiating cells in favor of neural crest specification and determining cell fate.^28^ It can also activate alpha-1-acid glycoprotein in mammalian livers as a transcriptional regulator.^29^ In addition, human NOLC1 is a binding target of doxorubicin, a widely used anti-cancer drug,^30^ and can also bind to and inhibit the catalytic subunit of CK2 *in vitro*.^31^ NOLC1 localizes to nucleolar dense fiber components (DFCs) and participates in the regulation of rRNA transcription by interacting with the largest subunit of RNA polymerase I (RPA194).^32^ Enhanced NOLC1 regulates the distributions of some nucleolar proteins such as NOG1, and thus disturbs rRNA processing.^33^

Telomeric repeat-binding factor 2 (TRF2) coats the length of all human telomeres^34^ by directly binding to duplex TTAGGG repeats, with >100 copies estimated to be present per chromosome end. The protection of human telomeres is dependent on this factor, and it is reasonable to assume that the requirement for TTAGGG repeats at chromosome ends reflects the need for TRF2 binding. Expression of a TRF2 mutant that blocks the accumulation of wild-type TRF2 on chromosome ends results in a deleterious phenotype similar to that induced by TRF2 inhibition.^35,36^ The cellular consequences of TRF2 inhibition suggest that TRF2-depleted telomeres are perceived as sites of DNA damage.^37^ TRF2 inhibition leads to induction of apoptosis and cell cycle arrest.^37^

It is reported that TRF2 localizes in the nucleolus of human MCF7 cells in a cell cycle-dependent manner; however, the underlying mechanism remains unclear.^38,39^ Here, we found that NOLC1 interacted with TRF2 and regulated its nucleolar retention.

## Results

### NOLC1 interacts with TRF2

**Figure 1:**
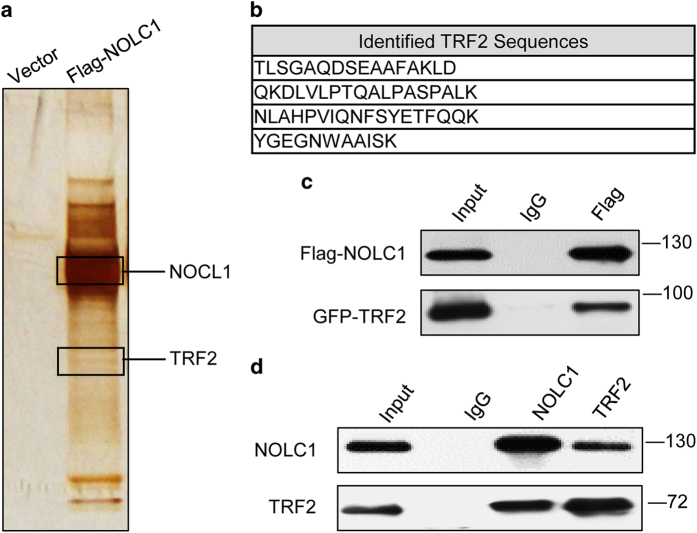
NOLC1 interacts with TRF2 in 293T cells. (**a**) *In vivo* Flag-tag pull-down analysis. Whole cell extracts of 293T cells transfected with Flag-NOLC1 or Flag-tagged-mock were immunoprecipitated with anti-Flag beads followed by mass spectrometric peptide sequencing. Both NOLC1 and TRF2 were identified. (**b**) MS identified TRF2 peptides. (**c**) *In vivo* binding of NOLC1 and TRF2. Lysates of 293T cells transfected with Flag-NOLC1 and GFP-TRF2 were immunoprecipitated with an anti-Flag monoclonal antibody and subjected to western blotting with anti-Flag and anti-GFP antibodies. (**d**) Reciprocal examination of the physical interaction between NOLC1 and TRF2. Immunoprecipitates obtained using an anti-TRF2 or anti-NOLC1 antibody were subjected to western blotting using anti-NOLC1 and anti-TRF2 antibodies.

NOLC1 was first identified as a nuclear localization signal-binding protein, and also functions as a chaperone for shuttling between the nucleolus and cytoplasm. To explore the potential proteins that interact with NOLC1, exogenous Flag-NOLC1 was pulled down and NOLC1-associated proteins were identified by mass spectrometry (MS) analysis (Figures 1a and b). Interestingly, TRF2 was identified. TRF2 binds to telomeres and functions in telomere protection,^11^ raising the possibility that NOLC1 participates in telomere-associated biological regulation. Confirming these MS results, when 293T cells were co-transfected with Flag-NOLC1 and GFP-TRF2, TRF2 was detected in the immunoprecipitate obtained using an anti-Flag antibody (Figure 1c). In addition, endogenous TRF2 was detected in the immunoprecipitate obtained from a 293T cell lysate using an anti-NOLC1 antibody (Figure 1d). Conversely, endogenous NOLC1 was detected in the immunoprecipitate obtained using an anti-TRF2 antibody (Figure 1d). Our results demonstrated that NOLC1 interacted with TRF2, which is consistent with the previous result that NOLC1 is found in the TRF2-interacting protein pool in 293T cells using a MS dual-tag TRF2 affinity purification system.^40^

### TRF2 and NOLC1 co-localize in the nucleolus

**Figure 2:**
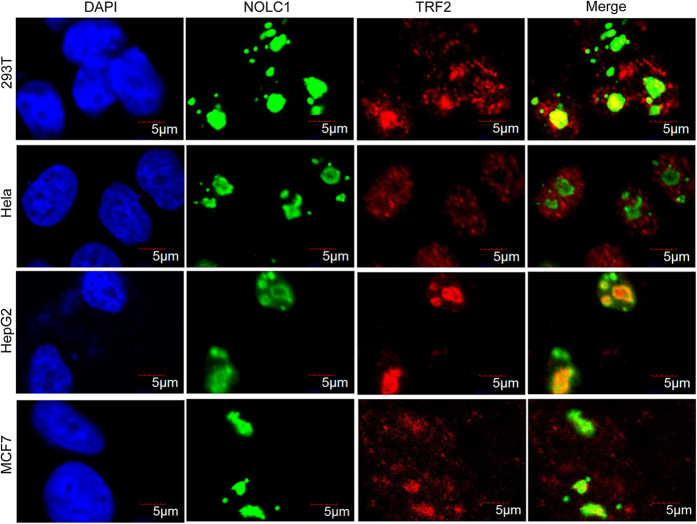
NOLC1 and TRF2 co-localize in the nucleolus of 293T, HepG2, and MCF7 cells, but not of Hela cells. Immunofluorescence analysis with a rabbit antibody against NOLC1 and a mouse antibody against TRF2 revealed nucleolar co-localization of TRF2 and NOLC1 in human 293T (first line), HepG2 (third line), and MCF7 (last line) cells, but not in Hela cells (second line). NOLC1 is shown in green. TRF2 is shown in red. Nuclei were visualized by DAPI staining. The localization of GFP-tagged TRF2 in Hela cells is shown in Supplementary Figure S1.

NOLC1 localizes in DFCs of the nucleolus.^32,33^ Considering the previous finding that TRF2 accumulates in the nucleolus,^38,39^ we hypothesized that NOLC1 may co-localize with TRF2. Indeed, immunofluorescence studies with the indicated antibodies against NOLC1 and TRF2 revealed that a significant portion of TRF2 localized in the nucleolus and co-localized with NOLC1 in human 293T, MCF7, and HepG2 cells (Figure 2). However, we failed to visualize TRF2 in the nucleolus of human Hela cells using the same antibody; instead, discrete foci were stained in the nucleoplasm, which probably correspond to telomeres (Figure 2). Exogenous GFP-TRF2 also failed to localize in the nucleolus (Supplementary Figure S1), similar to endogenous TRF2 in Hela cells. Furthermore, the localization pattern of NOLC1 was the same as that of TRF2 throughout the cell cycle (Supplementary Figure S2).

### NOLC1 does not influence TRF2 expression

**Figure 3:**
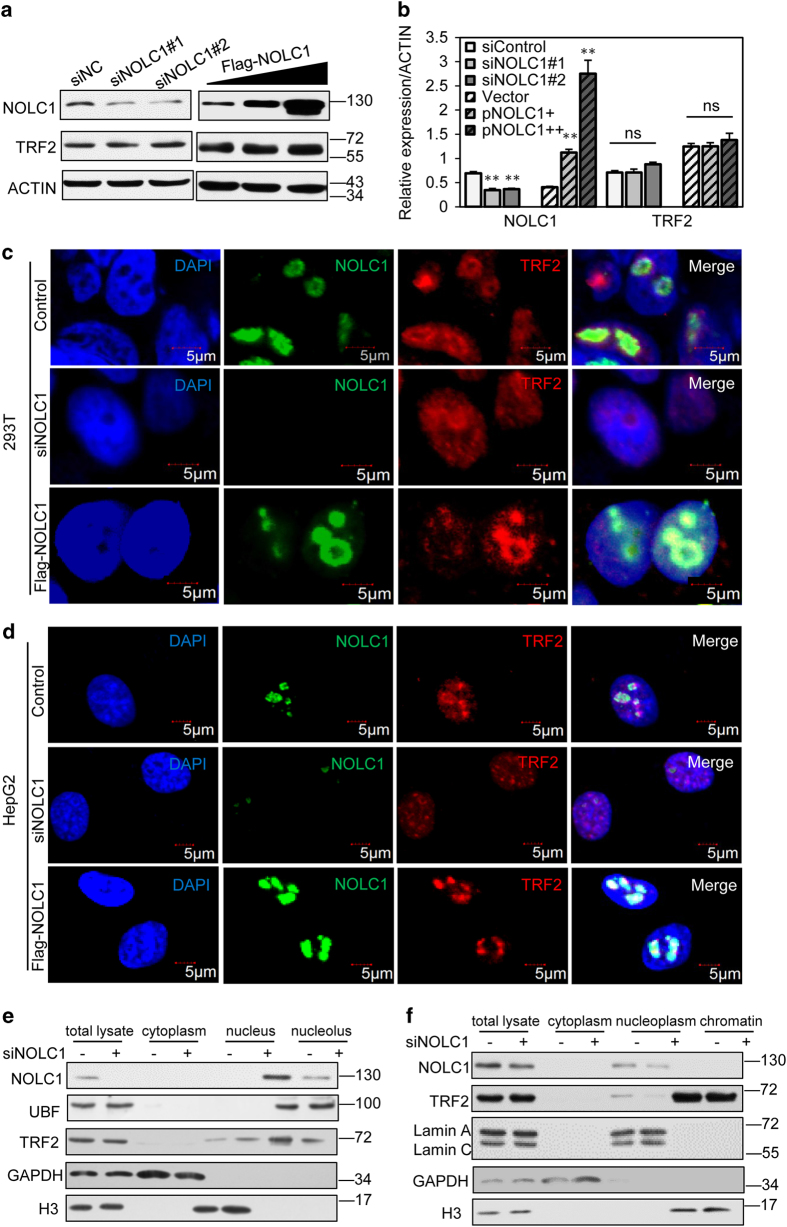
NOLC1 mediates the nucleolar accumulation of TRF2, but does not affect its expression. (**a**) HepG2 cells were transfected with NOLC1-targeting siRNA or Flag-NOLC1. Total cell lysates were subjected to western blot analysis with an anti-TRF2 mouse monoclonal antibody and an anti-NOLC1 rabbit monoclonal antibody. (**b**) Relative expression levels of NOLC1 and TRF2 proteins. ns, not significant. Data are presented as the mean±S.D. of three independently performed experiments. **P<0.01. (**c** and **d**) Localizations of NOLC1 and TRF2 in 293T and HepG2 cells transiently expressing Flag-NOLC1 or transfected with NOLC1-targeting siRNA. Immunofluorescence analysis was performed using the indicated antibodies to investigate the distribution of TRF2 protein and to determine the efficiency of NOLC1 knockdown or overexpression. NOLC1 is shown in green. TRF2 is shown in red. Nuclei were visualized by DAPI staining. (**e**) Sucrose gradient centrifugation revealed that the level of TRF2 was reduced in nucleolar extracts of human cells. Western blotting was performed to detect NOLC1, TRF2, UBF, GAPDH, and H3 in fractions of HepG2 cell nucleolar extracts. (**f**) HepG2 cells were transfected with control siRNA or NOLC1 siRNA for 72 h, chromatin extracts were subjected to western blot analysis with indicate antibodies.

It is reported that some nucleolar proteins such as NS and GNL3L modulate TRF1 and have antagonistic effects on TRF1 stability.^39^ Thus, we investigated if NOLC1 also affects the expression or stability of TRF2. Western blotting indicated that knockdown or overexpression of NOLC1 did not significantly affect expression of TRF2 (Figures 3a and b).

### NOLC1 mediates accumulation of TRF2 in the nucleolus

Increasing studies have revealed that nucleolar proteins such as PRL11 regulate the nucleolar retention of some proteins and thus affect their functions. We next examined whether NOLC1 modulated the localization of TRF2. 293T cells transfected with NOLC1-targeting small interfering RNA (siRNA) were analyzed by fluorescence microscopy. Cells were co-labeled with DAPI and for NOLC1 and TRF2 (Figure 3c). After NOLC1 knockdown in 293T cells, TRF2 was released from the nucleolus into the nucleoplasm. Similarly, NOLC1 knockdown induced the relocation of TRF2 from the nucleolus to the nucleus, increased the number of nucleoplasmic TRF2 foci, and enhanced the fluorescence intensity of these foci in human HepG2 cells. Exogenous expression of NOLC1 increased the nucleolar localization of TRF2, while the number of TRF2 foci in the nucleoplasm was decreased (Figure 3d). In addition, we isolated and extracted the nucleoli and analyzed the relative amount of TRF2 (Figure 3e). This suggested that the nucleolar retention of TRF2 was decreased while the nuclear distribution of TRF2 was increased after NOLC1 knockdown. Additionally, we extracted chromatin and analyzed the binding of TRF2 by western blotting after knockdown of NOLC1 (Figure 3f). The relative amount of TRF2 in the nucleoplasm (including the nucleolus) decreased after NOLC1 knockdown. However, we did not observe a significant increase in chromatin binding, which may be due to the relative abundance of TRF2 on telomeres. Taken together, our results demonstrated that NOLC1 participates in regulation of the nucleolar/nuclear distribution of TRF2.

### Nucleolar retention of TRF2 induces telomeric DNA damage

**Figure 4:**
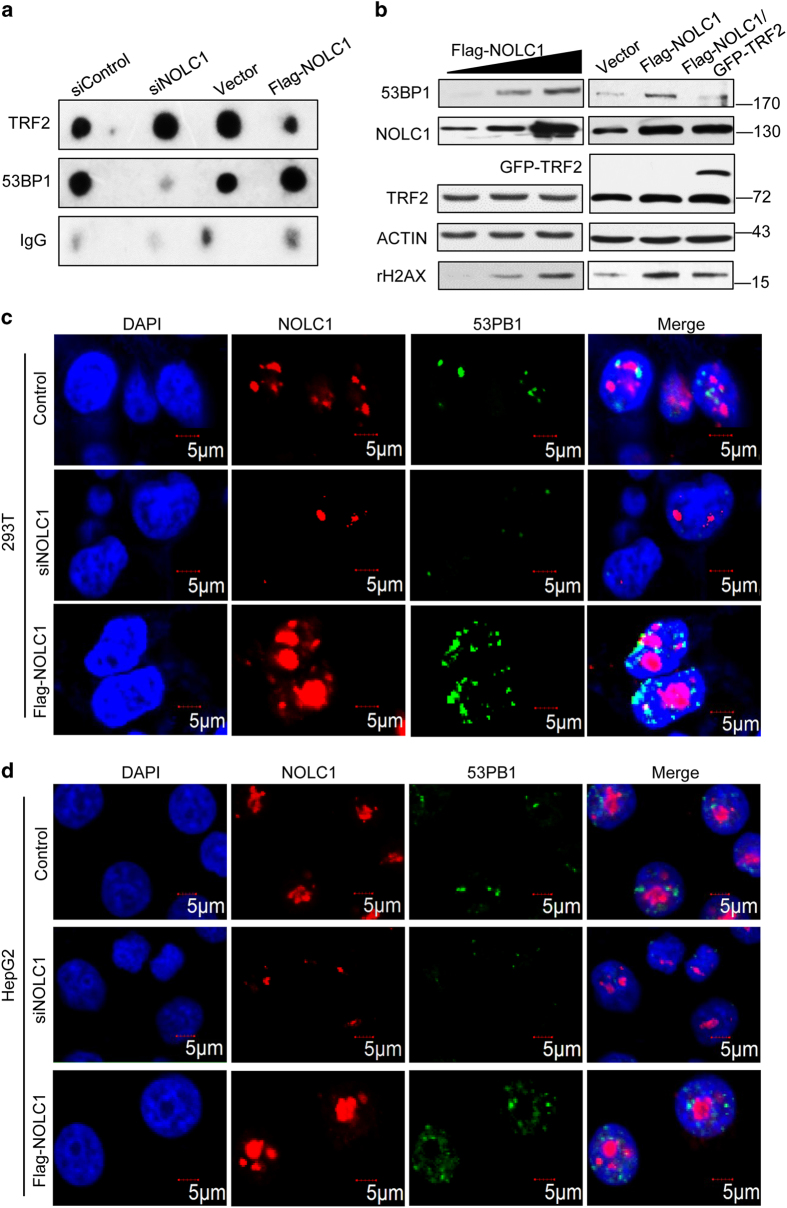
TRF2 shuttles to the nucleolus and induces the DNA damage response. (**a**) HepG2 cells were transfected with control or NOLC1-targeting siRNA for 72 h. Chromatin extracts were subjected to western blot analysis with the indicated antibodies. (**b**) The levels of 53BP1 and rH2AX were determined by western blotting in HepG2 cells transfected with Flag-NOLC1 or both Flag-NOLC1 and GFP-TRF2. (**c** and **d**) 53BP1 foci (green) and NOLC1 (red) in nuclei of 293T and HepG2 cells were visualized with the indicated antibodies after NOLC1 knockdown or overexpression. Nuclei were visualized by DAPI staining.

TRF2 specifically binds to the duplex (TTAGGG)n in telomeres and protects them from the DNA damage response.^41^ Given that overexpression of NOLC1 enhanced accumulation of TRF2 in the nucleolus, we next investigated if the nucleolus-nucleoplasm shuttling of TRF2 affects its binding to telomeres. We analyzed the binding of TRF2 to telomeres by chromatin immunoprecipitation (ChIP). As expected, the relative amount of TRF2 on telomeres increased after NOLC1 knockdown, but decreased upon NOLC1 overexpression (Figure 4a upper). Interestingly, telomere binding of 53BP1, which is a DNA damage response protein, decreased after NOLC1 knockdown, but increased upon NOLC1 overexpression (Figure 4a middle). This raised the question of whether the decreased level of TRF2 on telomeres upon NOLC1 overexpression induces telomeric DNA damage. TRF2 is a component of the shelterin complex, which also consists of TRF1, RAP1, POT1, TPP1, and TIN2.^42^ Knockdown of TRF2 or expression of a dominant-negative TRF2 mutant lacking the telobox sequence and N-terminal basic domain (TRF2^DBDM^) can induce telomere damage and formation of 53BP1 and rH2AX foci.^42^ To further address this question, we analyzed the activities of 53BP1 and rH2AX upon NOLC1 overexpression. Western blotting demonstrated that 53BP1 and rH2AX activities increased upon NOLC1 overexpression (Figure 4b left) and this was rescued by co-expression of TRF2 (Figure 4b right). Fluorescence microscopic analysis confirmed that the number of 53BP1 foci was decreased after NOLC1 knockdown in both 293T and HepG2 cells, but increased upon NOLC1 overexpression (Figures 4c and d), similar to the effect of etoposide treatment (Supplementary Figure S3).

### Heterogenous expression of NOLC1 induces apoptosis, and this is rescued by co-expression of TRF2

**Figure 5:**
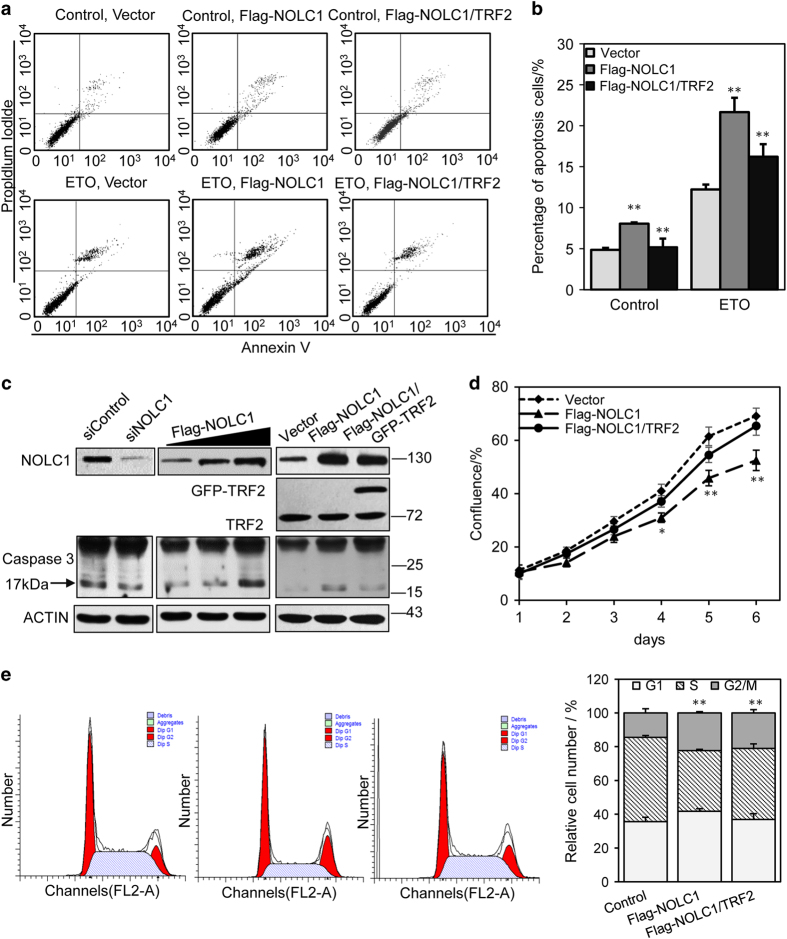
Overexpression of NOLC1 promotes apoptosis and cell cycle arrest. (**a**) Parametric dot plots showing annexin V *versus* PI staining in HepG2 cells transfected with the mock vector, Flag-NOLC1, or both Flag-NOLC1 and TRF2 for 24 h. For etoposide treatment, cells were treated with 40 *μ*M etoposide for 24 h before being collected for flow cytometric analysis. (**b**) Each histogram represents the percentages of apoptotic cells. (**c**) Western blotting was performed to investigate caspase 3 activity after NOLC1 was knocked down or overexpressed in HepG2 cells. (**d**) The HepG2 cell growth rate was monitored after NOLC1 and TRF2 overexpression. (**e**) Cell cycle analyses following PI labeling showed that NOLC1 overexpression increased the percentages of HepG2 cells in G0/G1 and G2/M, while overexpression of TRF2 rescued the cell cycle arrest induced by NOLC1 overexpression. Data are presented as the mean±S.D. of three independently performed experiments. *P<0.05; **P<0.01.

Telomere dysfunctions such as the failed binding of TRF2 to telomeres can induce a DNA damage response pathway, apoptosis for instance. Therefore, we examined whether NOLC1 overexpression is associated with apoptosis. Flow cytometric analysis revealed that, after transfection of NOLC1 for 48 h, 8.04% of NOLC1-overexpressing HepG2 cells were apoptotic compared with 4.85% of untreated cells. Meanwhile, the relative level of apoptotic cells was lower upon co-transfection of NOLC1 and TRF2 than upon transfection of NOLC1 alone (Figures 5a and b). Moreover, upon etoposide treatment, 21.85% of NOLC1-overexpressing cells were apoptotic compared with 12.21% of control cells. Western blotting also showed that expression of the apoptosis marker caspase 3 increased upon NOLC1 overexpression and this was rescued by TRF2 overexpression (Figure 5c). This observation further corroborated the idea that NOLC1 overexpression induces apoptosis. However, the increase in apoptosis was not as marked as that induced by TRF2 knockdown or expression of truncated TRF2. This may be because the redistribution of TRF2 from telomeres to the nucleolus upon NOLC1 overexpression was more subtle than the dramatic decrease in binding of TRF2 to telomeres upon TRF2 knockdown or expression of truncated TRF2.

### NOLC1 overexpression suppresses cell proliferation

Several lines of evidence support the idea that TRF2 dysregulation induces telomeric DNA damage and thus restrains cell proliferation and induces apoptosis. To further investigate whether the NOLC1-mediated relocalization of TRF2 from telomeres to the nucleolus influences cell proliferation, fluorescence-activated cell sorting analysis was carried out following NOLC1 knockdown or overexpression. The cell cycle distributions of HepG2 cells treated with scrambled or NOLC1-targeting siRNA for 72 h are shown in Figures 5d and e. NOLC1 knockdown decreased the percentage of cells in G0/G1 phase. Conversely, NOLC1 overexpression increased the percentage of cells in G0/G1 phase, while TRF2 overexpression rescued the cell cycle arrest and cell proliferation inhibition induced by NOLC1 overexpression. This is consistent with the previous finding that TRF2 inhibition arrests the cell cycle in G0/G1 phase.

## Discussion

The nucleolus is the most prominent compartment in the nucleus and is the site of ribosome biogenesis in eukaryotes. Recently, many non-traditional functions of the nucleolus have been proposed.^9–13^ These include signal recognition particle assembly, small RNA modification, RNA editing, telomerase maturation, nuclear export, cell cycle control, and stress sensing. Some telomere-related proteins such as telomerase and TRF1 have been reported to localize in the nucleolus or to interact with nucleolar proteins.^12,20^

Human TRF2 localizes in the nucleolus; however, the underlying molecular mechanism remains unclear. Here, we found that TRF2 interacted with the nucleolar protein NOLC1. Confocal microscopy indicated that TRF2 co-localized with NOLC1 in the nucleoli of human 293T, MCF7, and HepG2 cells, but not of Hela cells. Considering the lower expression of NOLC1 in Hela cells (Supplementary Figure S4A), we overexpressed NOLC1 in these cells (Supplementary Figure S4B). NOLC1 and TRF2 co-localized in these cells, although this was not as apparent as in 293T or HepG2 cells. Knockdown or overexpression of NOLC1 did not affect expression of TRF2, but influenced its nucleolar accumulation. Ablation of NOLC1 expression promoted the release of nucleolar TRF2 into the nucleoplasm, and the number of nucleoplasmic TRF2 foci was increased in both HepG2 and 293T cells. Conversely, NOLC1 overexpression mediated the accumulation of TRF2 in the nucleolus and decreased its nucleoplasmic level. In addition, chromatin isolation and ChIP demonstrated that the nucleoplasmic level of TRF2 and its telomere binding were decreased upon NOLC1 overexpression. Telomeres are essential chromosomal elements comprising 2–20 kb of double-stranded TTAGGG repeats that ensure proper replication and protection of chromosome ends. The shelterin subunit TRF2 plays an essential role in protecting chromosome ends by facilitating their organization into the protective capping structure.^42,43^ Functional inhibition of TRF2 induces uncapped or dysfunctional telomeres, which is characterized by loss of telomere overhangs and DNA damage responses at telomeres,^41,44^ leading to cell cycle arrest and apoptosis. Given that NOLC1 modulated TRF2 shuttling between the nucleolus and telomere, we investigated if this influences the DNA damage response. NOLC1 overexpression enhanced the activities of 53BP1 and rH2AX as well as the binding of 53BP1 to telomeres. In addition, the number of 53BP1 foci was decreased upon NOLC1 inhibition and increased after NOLC1 overexpression in both 293T and HepG2 cells. Moreover, the percentage of apoptotic cells was higher among NOLC1-overexpressing cells than among control cells. Cell cycle progression was arrested after heterogenous expression of NOLC1. Furthermore, co-expression of TRF2 rescued NOLC1 overexpression-induced cell proliferation inhibition and cell cycle arrest.

**Figure 6:**
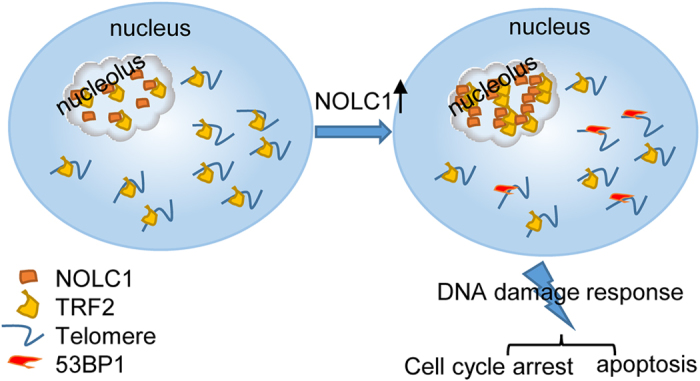
A proposed working model to explain how NOLC1 functions in the nucleolar accumulation of TRF2. Under normal conditions, the levels of TRF2 in the nucleolus and at telomeres are strictly balanced. When NOLC1 expression increases, TRF2 accumulates in the nucleolus and disassociates from telomeres, leading to the DNA damage response at telomeres, which promotes apoptosis and cell cycle arrest.

The nucleolar accumulation of some proteins such as MDM2 is regulated by more than one nucleolar protein. In this study, the nucleolar localization of TRF2 was not absolutely with NOLC1. Other nucleolar proteins may also participate in the nucleolar retention of TRF2, although this requires further investigation. In addition, it would be interesting to investigate whether other telomere-related proteins are also regulated by nucleolar proteins, in addition to telomerase and TRF2. In summary, this study demonstrated that the nucleolar protein NOLC1 interacted with the telomere-binding protein TRF2 and mediated its nucleolar accumulation. We also found that NOLC1 overexpression decreased the binding of TRF2 to telomeres, induced a DNA damage response at telomeres, and thus promoted apoptosis and cell cycle arrest (Figure 6). Our research suggests a potential way via which the nucleolus regulates the function of TRF2 on telomeres.

## Materials and methods

### Cell culture, antibodies, and plasmids

The human embryonic kidney cell line HEK293T, the human breast cancer cell line MCF7, the human hepatoma cell line HepG2, and Hela cells were cultured in Dulbecco’s modified Eagle’s medium supplemented with 10% fetal bovine serum, 100 units/ml penicillin, and 100 *μ*g/ml streptomycin in 5% CO_2_ at 37 °C. The antibodies used in this study were as follows: anti-NOLC1 (ab184550, Abcam, Cambridge, MA, USA; and sc-374033, Santa Cruz Biotechnology, Dallas, TX, USA), anti-TRF2 (ab13579, Abcam), anti-UBF (sc-9131, Santa Cruz Biotechnology), anti-Lamin A/C (sc-6215, Santa Cruz Biotechnology), anti-GAPDH (5632-1, Epitomics, Cambridge, MA, USA), anti-Flag (F1804, Sigma, St Louis, MO, USA), anti-H3 (AM8433, ABGENT, San Diego, CA, USA) and anti-actin (SC-130300, Santa Cruz Biotechnology). The Flag-NOLC1 expression vector was constructed by inserting the full-length NOLC1 cDNA into the pCMV-Flag vector. The GFP-TRF2 expression vector was purchased from Addgene (ID no. 19798).

### Flag-tag affinity purification of NOLC1 and associated proteins

MS was performed as described previously.^45^ Briefly, 293T cells were seeded on four 15 cm culture dishes, grown to 60% confluency, and transfected with Flag-NOLC1 for 48 h. Cells were collected and proteins were extracted by a freeze/thaw lysis procedure that aims to keep the lysate as concentrated as possible. The crude lysate was precleared by centrifugation, and the bait proteins and their associated proteins were affinity purified using the Flag-tag and then eluted using the Flag peptide. The eluates were resolved by sodium dodecyl sulfate (SDS)-polyacrylamide gel electrophoresis (PAGE) and visualized by silver staining. The protein bands were retrieved and analyzed by MS.

### Immunofluorescence

Cells were grown on coverslips and fixed for 10 min in 4% paraformaldehyde. After being washed twice in phosphate-buffered saline (PBS) for 5 min, cells were incubated for 5 min in PBS containing 0.5% Triton X-100. Cells were blocked in 5% bovine serum albumin prepared in PBST, incubated overnight with primary antibodies prepared in blocking solution, and then incubated with a secondary antibody conjugated with 488-Alexa (green) or 647-Alexa (red) (Molecular Probes, Eugene, OR, USA). DAPI was used as a nuclear stain. Microscopic analyses were performed using an Olympus FV1500 confocal microscope.

### Immunoprecipitation and western blotting

Cells were collected in immunoprecipitation lysate buffer (50 mM Tris-HCl (pH 7.4), 150 mM NaCl, 1 mM EDTA, 1 mM DTT, 0.25 mM PMSF, 0.3% NP40, and a cocktail). The lysates were incubated with appropriate antibodies overnight at 4 °C and then with protein-G Sepharose beads (Millipore, Darmstadt, Germany) for another 2 h at 4 °C. The immunoprecipitates were washed four times with immunoprecipitation lysate buffer and resuspended in 30 μl of 2× SDS loading buffer. The samples were boiled for 10 min for western blot analysis. Proteins were extracted, separated by SDS-PAGE, and transferred to a nitrocellulose filter membrane. The membrane was blocked with 5% nonfat milk for 1 h at room temperature and incubated with primary antibodies overnight at 4 °C. After being washed twice, the membrane was incubated at room temperature for 1 h with a secondary antibody (1 : 5000). The bands were visualized using enhanced chemiluminescence. The western blot raw data was supplied in Supplementary Information-WB raw data.

### Plasmid and siRNA transfection

Plasmids were transfected using Lipofectamine 2000 transfection reagent (Invitrogen, Carlsbad, CA, USA) following the manufacturer’s instructions. To knock-down NOLC1, HEK293T or HepG2 cells were transfected with siRNA duplexes using RNAiMax transfection reagent (Invitrogen). The following siRNA sequences specific for NOLC1 were used: #1: 5′- CACCAAGAAUUCUUCAAAU-3′ and #2: 5′- GCGAAAGUUACAGGCAAAU-3′. Scrambled siRNA (5′- UUCUCCGAACGUGUCACGU-3′) was used as a control and did not target any known gene in the databases.

### Nucleolar extraction

Cellular nucleolar fractions were obtained as previously described.^37^ Briefly, pelleted cells were resuspended in ice-cold mild detergent buffer and centrifuged for 10 min at 1350 g. The supernatant was retained as the cytoplasmic fraction. The pellets were then resuspended in 0.25 M sucrose, layered over a cushion of 0.35 M sucrose, and centrifuged. The resulting pellet was resuspended in 0.35 M sucrose, sonicated for 45 s, layered over a cushion of 0.88 M sucrose, and centrifuged to pellet the nucleoli. The supernatant was retained as the nucleoplasmic fraction. The nucleoli were washed by resuspension in 0.5 ml of 0.35 M sucrose followed by centrifugation. The nucleolar pellet was resuspended in high-salt buffer, sonicated, and centrifuged. The supernatant was retained as the nucleolar extract, and the NaCl concentration was adjusted to 150 mM.

### Chromatin isolation

Chromatin isolation was carried out as described previously.^46^ Briefly, 2×10^6^ cells were washed with PBS, resuspended in 200 ml of solution A (10 mM HEPES, pH 7.9, 10 mM KCl, 1.5 mM MgCl_2_, 0.34 M sucrose, 10% glycerol, 1 mM DTT, 0.05% Triton X-100, 1× protease inhibitor cocktail, and 1× phosphatase inhibitor cocktail I and II), and incubated on ice for 5 min. Nuclei were collected by centrifugation at 14 000 r.p.m. for 5 min. Isolated nuclei were washed once with solution A and then lysed in solution B (3 mM EDTA, 0.2 mM EGTA, 1 mM DTT, 1× protease inhibitor cocktail, and 1× phosphatase inhibitor cocktail I and II) for 10 min on ice. Chromatin was collected by centrifugation (2000 r.p.m. for 4 min), washed once in solution B, and centrifuged again at 14 000 r.p.m. for 1 min. The final chromatin pellet was resuspended in Laemmli buffer and sonicated for 15 s.

### Cell cycle and apoptosis analyses

For cell cycle analysis, non-transfected and NOLC1-transfected HepG2 cells were cultured to 80% confluency, washed in PBS, trypsinized, fixed in 75% ice-cold ethanol overnight, and treated with 20 *μ*g/ml DNase-free RNase A at 37 °C for 30 min. The cells were stained with propidium iodide (PI; 50 *μ*g/ml; Sigma), and flow cytometry was conducted. Cell cycle profiles were analyzed using Multi Cycle AV software. In the apoptosis assay, cells (1×10^5^) from each sample were subjected to annexin V/PI staining (Dojindo, Kumamoto, Japan) according to the manufacturer’s instructions.

### Statistical analysis

The data were statistically analyzed using the *t* test. *P*<0.05 was considered to represent a statistically significant difference. Data are representative of three independent experiments performed in triplicate.

## Additional Information

**Publisher’s note** Springer Nature remains neutral with regard to jurisdictional claims in published maps and institutional affiliations.

## Notes

Supplementary Information

Supplementary Information – WB Raw Data
