# Supplementary material for: Nucleolar and coiled-body phosphoprotein 1 (NOLC1) regulates the nucleolar retention of TRF2
Source: Cell Death Discov. 2017 Sep 4;3:17043–. doi: 10.1038/cddiscovery.2017.43 (PMC5582526; doi:10.1038/cddiscovery.2017.43)
Supplement: Supplementary Information [file cddiscovery201743-s1.pdf]

Supplementary Information

Nucleolar and Coiled-body Phosphoprotein 1 (NOLC1) Regulates the Nucleolar Retention of TRF2

Fuwen Yuan[1], Guodong Li[1], and Tanjun Tong[1], *

[1] Research Center on Aging, Department of Biochemistry and Molecular Biology, School of Basic Medical Sciences, Peking University, Beijing, China.

* Correspondence and requests for materials should be addressed to T. T. (Email: ttj@bjmu.edu.cn)

Fig. S1

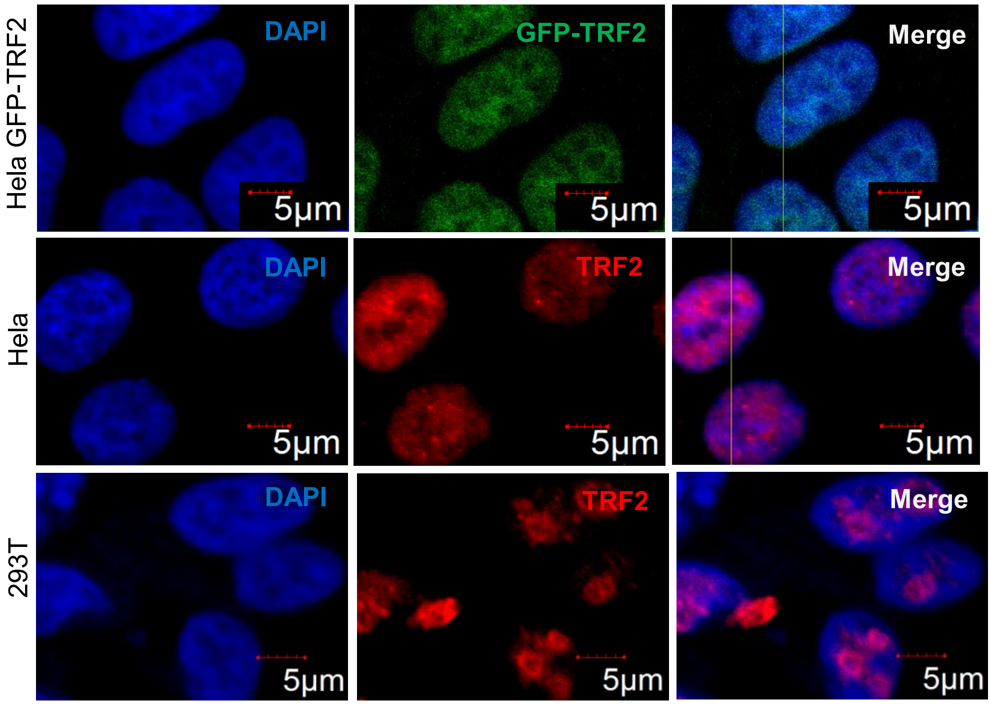

Fig. S1. The localization of TRF2 in Hela and 293T cells. Immunofluorescence with a mouse antibody against TRF2 or heterogenous expression GFP-tag TRF2 revealed a nucleus localization in human Hela (the first and second line), while located in nucleolus in 293T cells (the last line).

Fig. S2

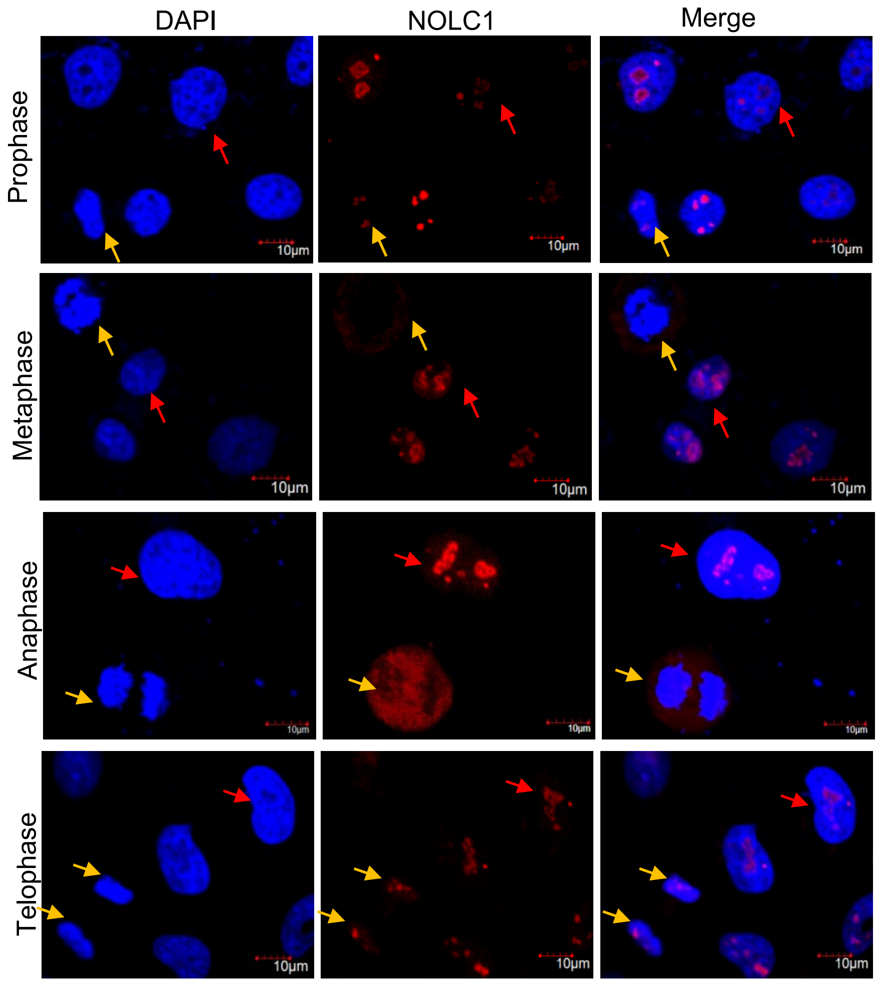

Fig. S2. NOLC1 shows a cell cycle dependent distribution in HepG2 cells. Immunofluorescence with a rabbit antibody against NOLC1 reveals a cell cycle depend localization of NOLC1 in human HepG2 cells. In metaphase, NOLC1 release to nucleus.

Fig. S3

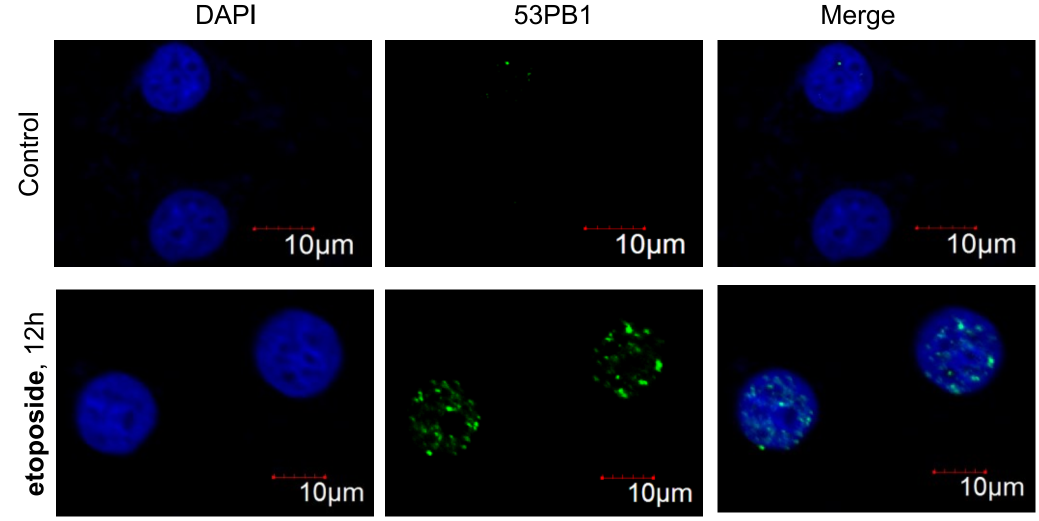

Fig. S3. 53BP1 was induced with etoposide treatment. HepG2 cells was treated with 40uM etoposide for 12 hours before harvest for analysis, immunofluorescence with a rabbit antibody against 53BP1 indicated that the 53PB1 foci was induced with etoposide treatment.

Fig. S4

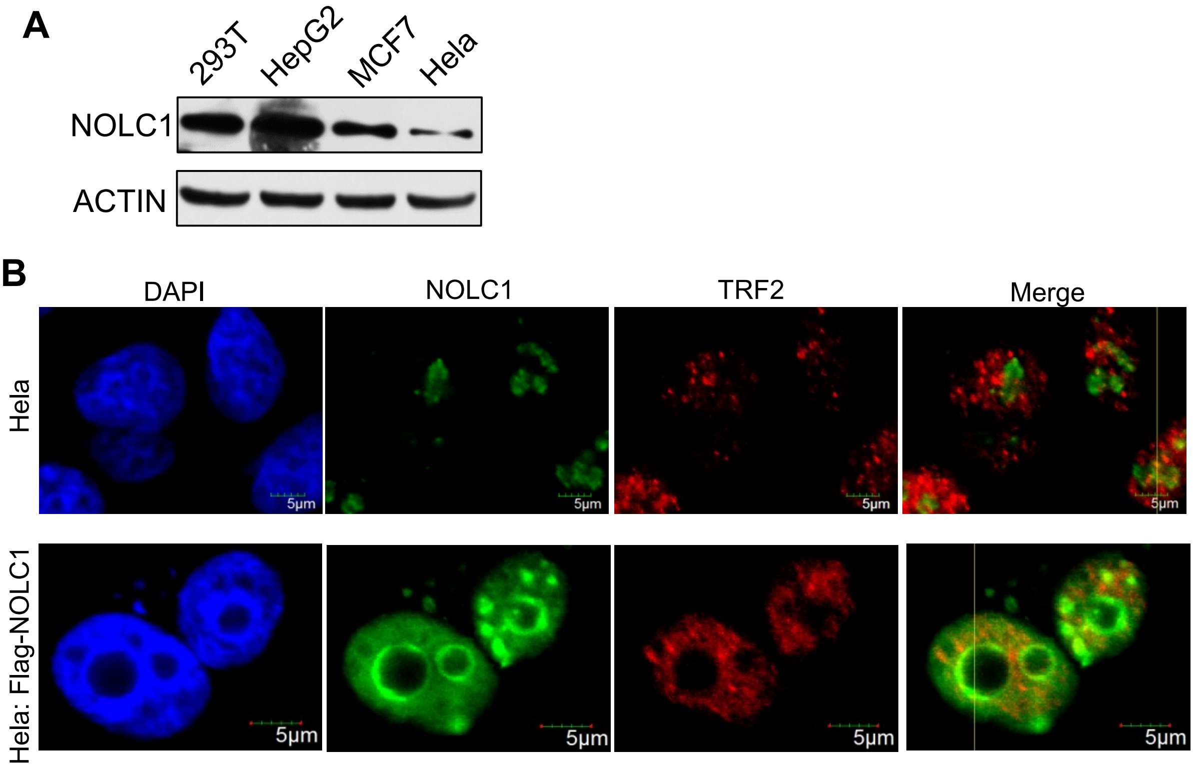

Fig. S4. NOLC1 have no significant influence on the distribution of TRF2 in Hela cells as in 293T or HepG2 cells. (A) The expression of TRF2 in 293T, HepG2, MCF7 and Hela cells was detected by western blot. (B) Hela cells were transfected with Flag-NOLC1 for 48 hours. Immunofluorescence with a rabbit antibody against NOLC1 and a mouse antibody against TRF2 reveals a weak co-localization of NOLC1 and TRF2 in human Hela cells.
