# Supplementary material for: Nucleolar and coiled-body phosphoprotein 1 (NOLC1) regulates the nucleolar retention of TRF2
Source: Cell Death Discov. 2017 Sep 4;3:17043–. doi: 10.1038/cddiscovery.2017.43 (PMC5582526; doi:10.1038/cddiscovery.2017.43)

WB, Figure 1c

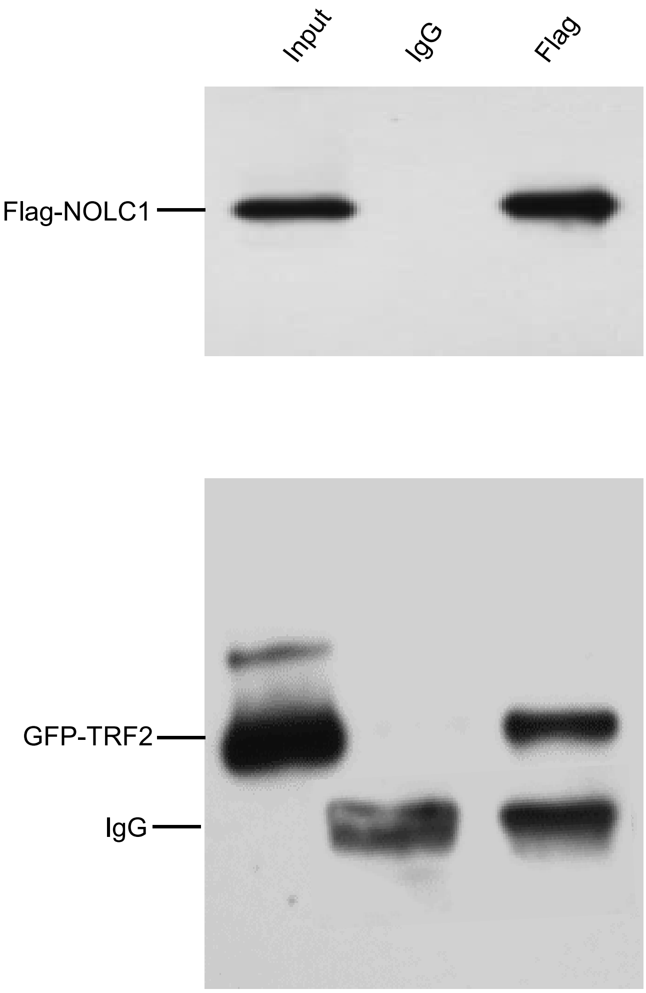

WB, Figure 1d

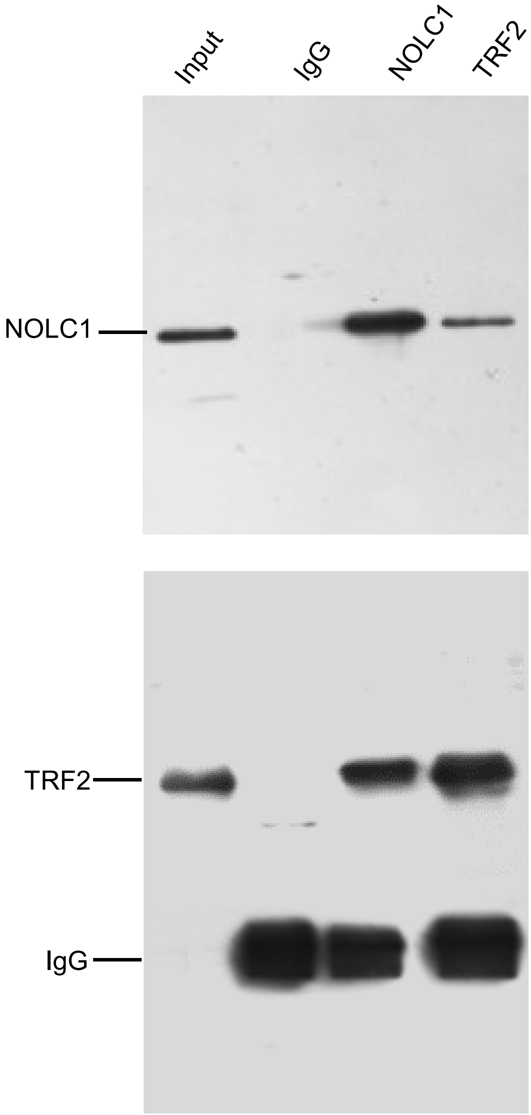

WB, Figure 3a

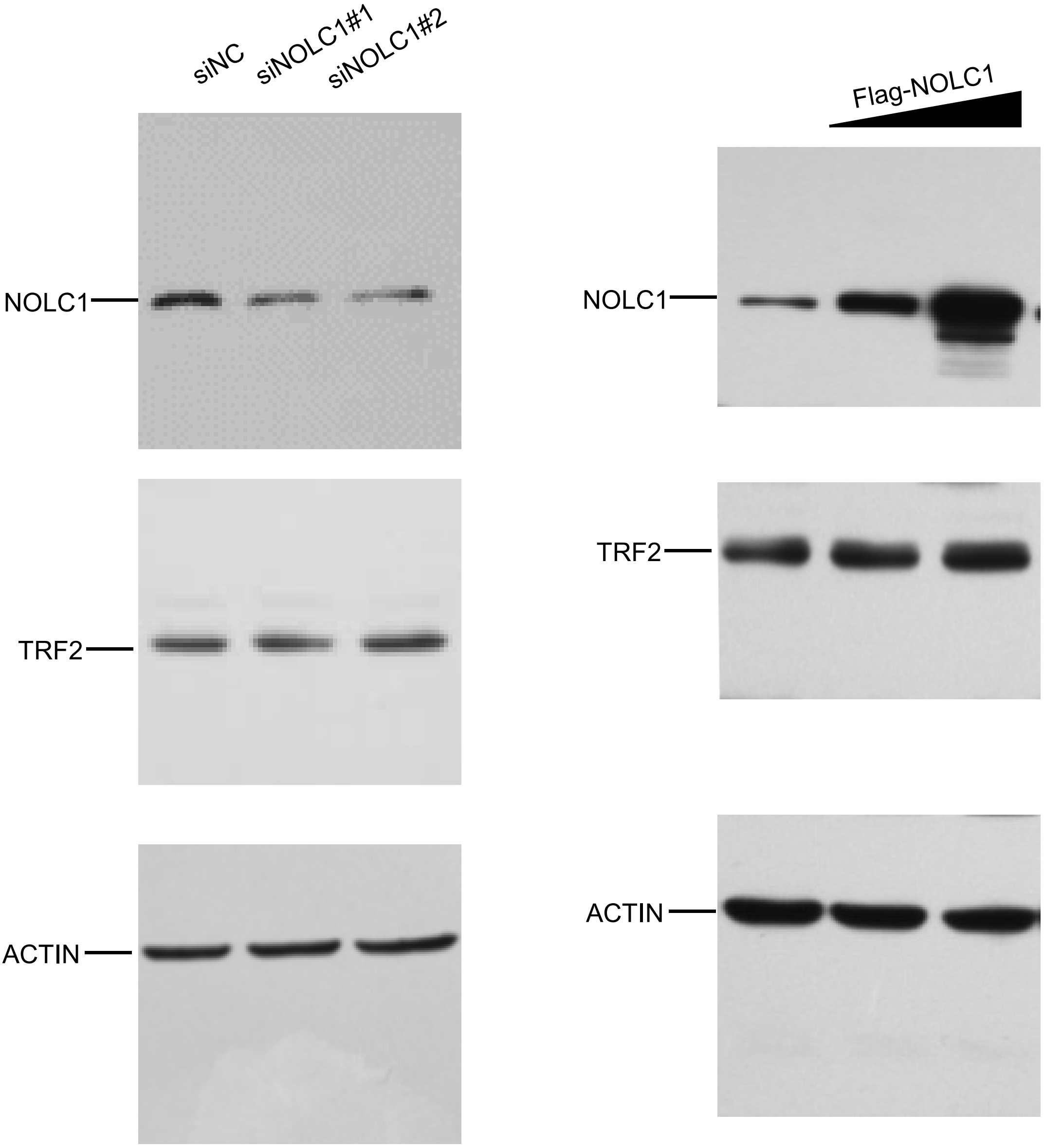
siNC
siNOLC1#1
siNOLC1#2
NOLC1
TRF2
ACTIN
Flag-NOLC1
NOLC1
TRF2
ACTIN

WB, Figure 3e

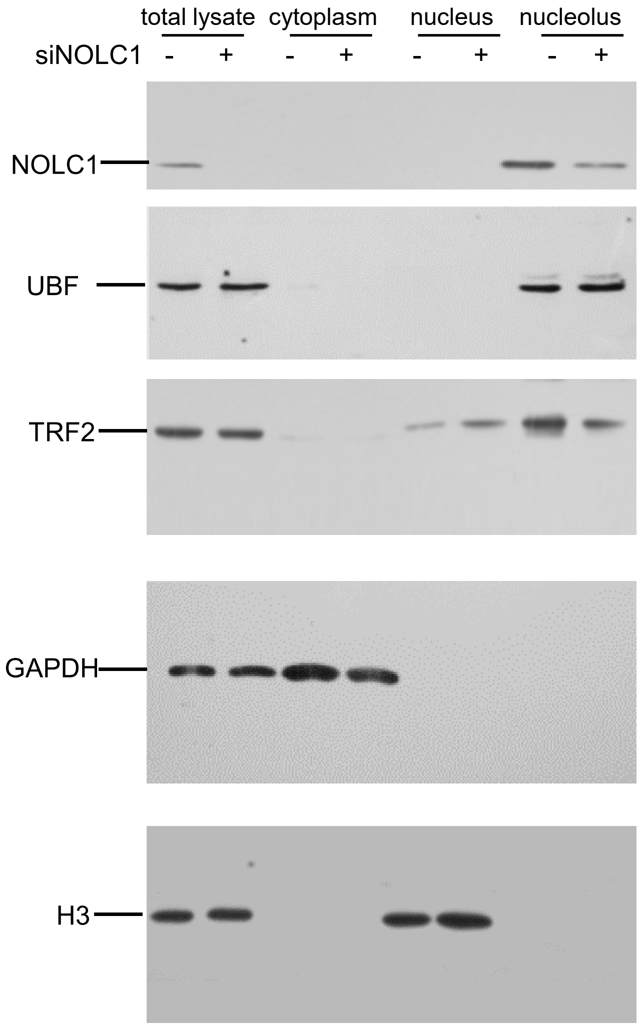

WB, Figure 3f

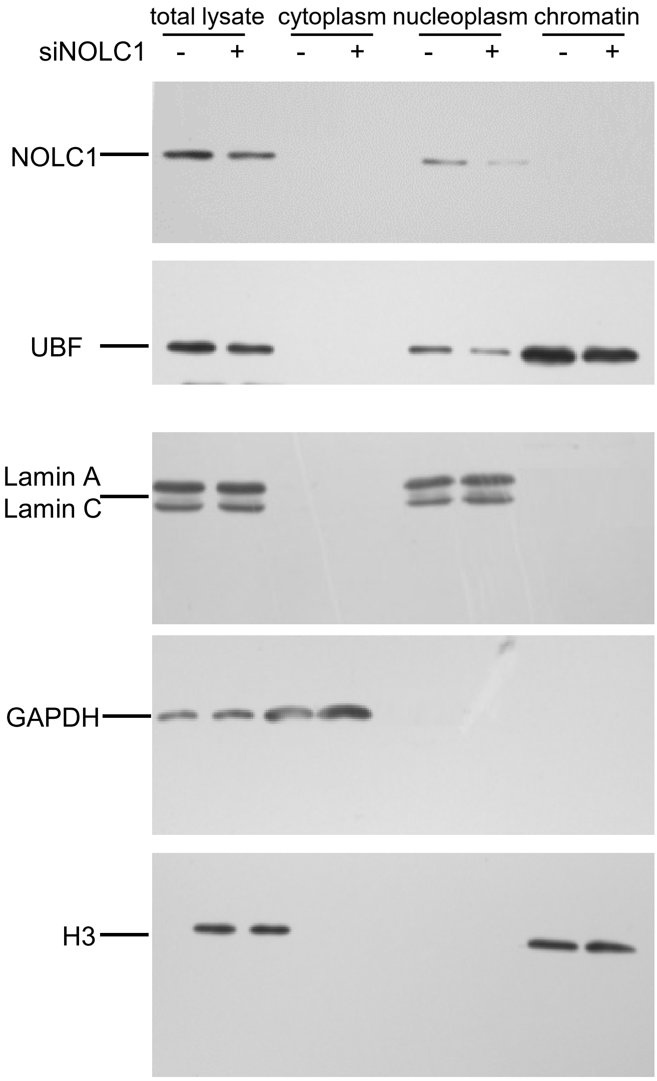

WB, Figure 4b

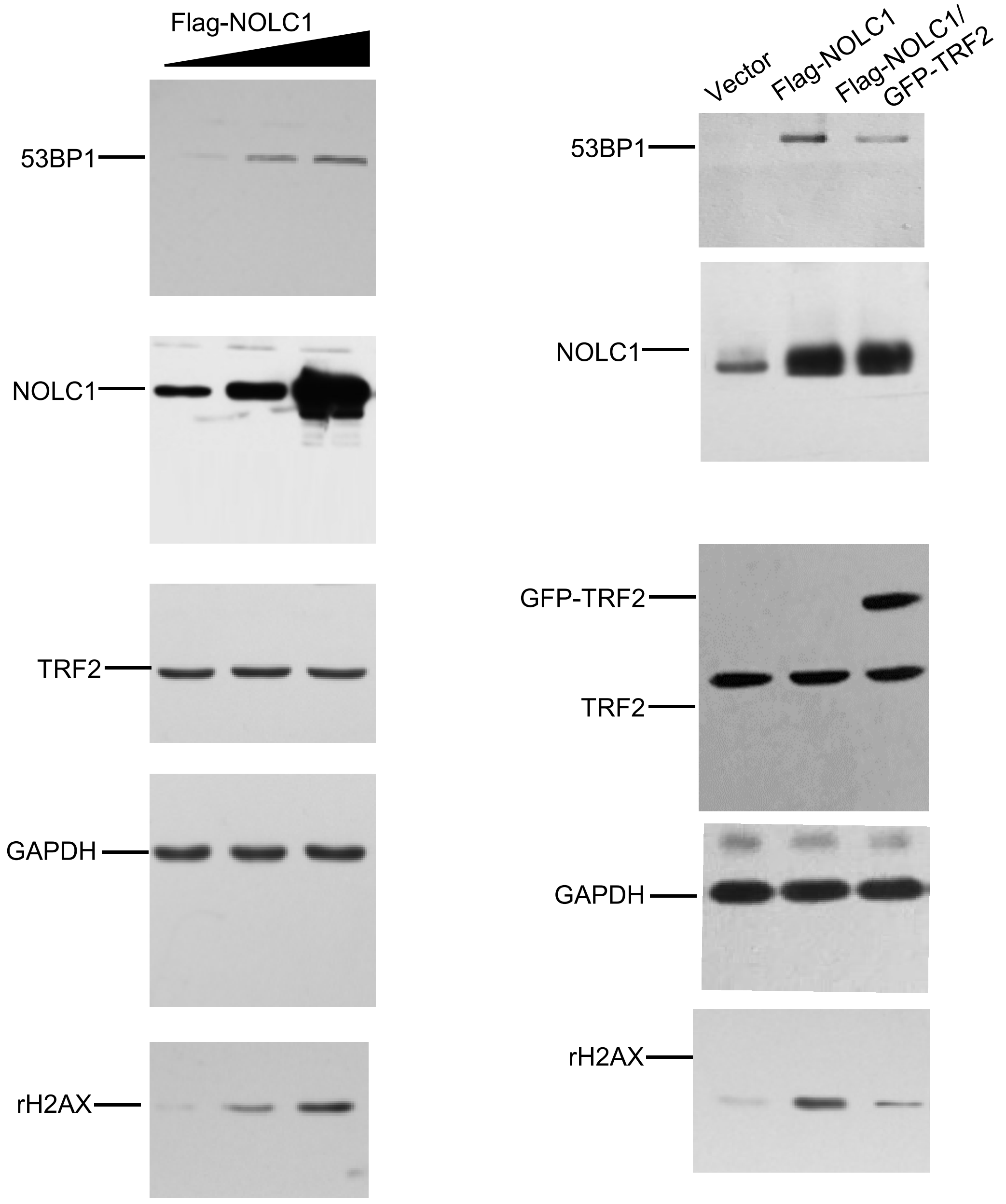
Flag-NOLC1
53BP1
NOLC1
TRF2
GAPDH
rH2AX
Vector
Flag-NOLC1
Flag-NOLC1/
GFP-TRF2
53BP1
NOLC1
GFP-TRF2
TRF2
GAPDH
rH2AX

WB, Figure 5c, left

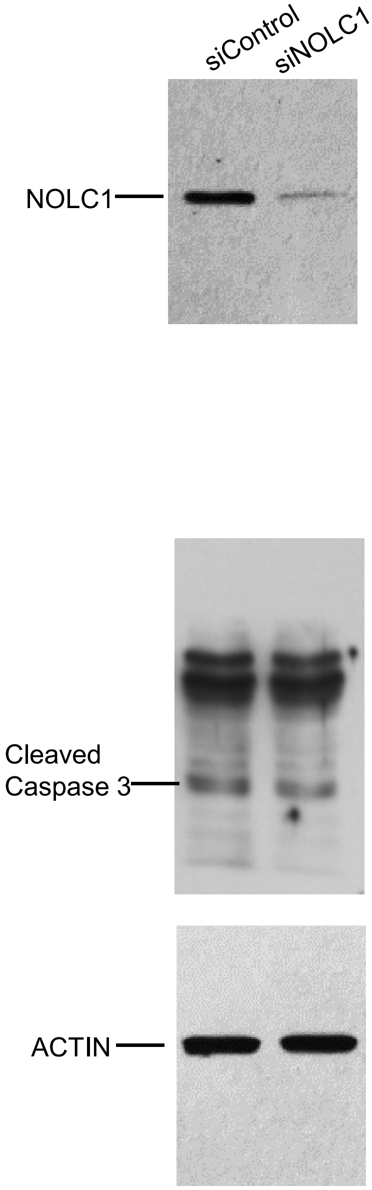

WB, Figure 5c, middle

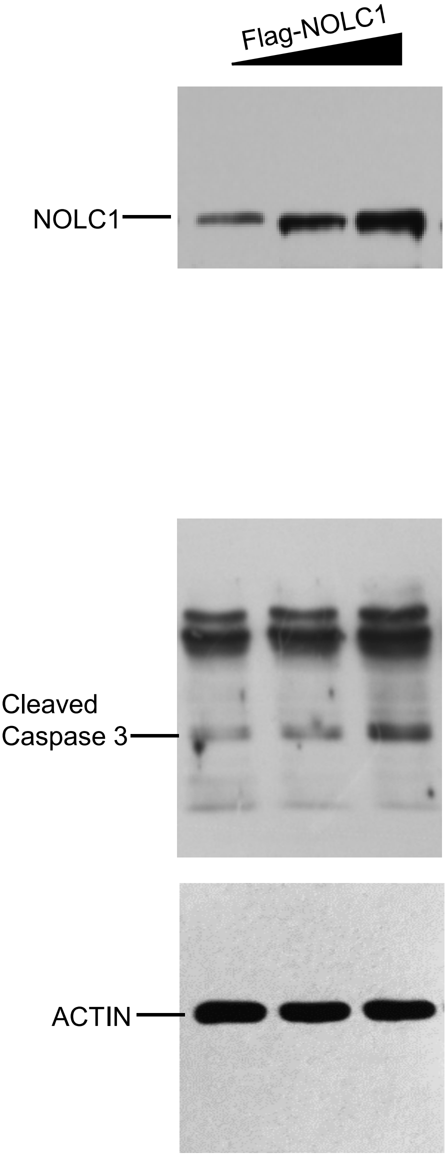

WB, Figure 5c, right

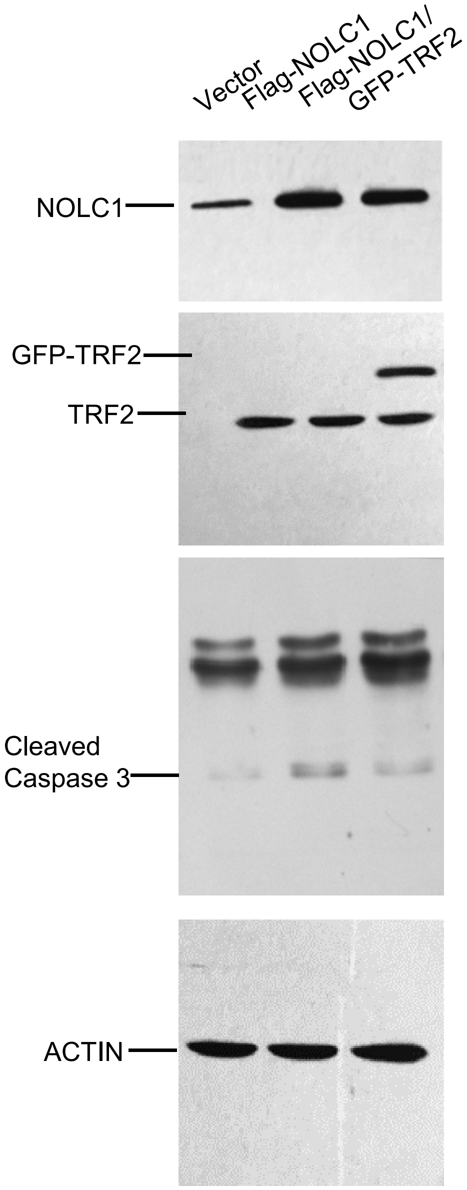

Supplement: Supplementary Information – WB Raw Data [file cddiscovery201743-s2.pdf]
